# Harnessing a Surface Water-Based Multifaceted Approach to Combat Zoonotic Viruses: A Rural Perspective from Bangladesh and China

**DOI:** 10.3390/microorganisms13112526

**Published:** 2025-11-04

**Authors:** Yizhe Wu, Yuqing Long, Xueling Yang, Xin Du, Xinyan Du, Nusrat Zahan, Zhiqiang Deng, Chen Du, Songzhe Fu

**Affiliations:** 1 School of Life Science, Northwest University, Xi’an 710069, China; wyzofficial@163.com (Y.W.); 18298434713@163.com (X.D.); 2 School of Medicine, Northwest University, Xi’an 710069, China; 15892663512@163.com (Y.L.); m18983265580@163.com (X.D.); 3 Ocean University of China, Qingdao 266100, China; yangxueling2001@163.com; 4 Department of Aquaculture, Chattogram Veterinary and Animal Sciences University, Chattogram 4202, Bangladesh; nusrat_zahan@126.com; 5 Nanchang Center for Disease Control and Prevention, Nanchang 330038, China; dengzhiqiang4540@163.com; 6 Shenzhen Center for Disease Control and Prevention, Shenzhen 518020, China; duchen199407@163.com

**Keywords:** avian influenza virus (AIV), dengue virus, rural regions, surface water-based surveillance (SWBS)

## Abstract

Rural tropical regions face escalating threats from zoonotic AIV and dengue virus but lack sewered infrastructure for conventional wastewater surveillance. We implemented surface water-based surveillance (SWBS) in peri-urban Dhaka (Bangladesh) and Ruili (China) from July to November 2023 and coupled it with machine learning-enhanced digital epidemiology. Reverse transcription quantitative PCR (RT-qPCR) was employed to detect the M gene of AIV and to subtype H1, H5, H7, H9, and H10 in surface water. Wild bird feces (*n* = 40) were collected within 3 km of positive sites to source-track AIV. For the dengue virus, a serogroup-specific RT-qPCR assay targeting the CprM gene was used. Genomic sequencing of AIV and dengue virus was performed to elucidate phylogenetic relationships with local clinical strains. Clinical data related to dengue fever were also collected for correlation analysis. Meanwhile, 13 dengue-related keyword search volumes were harvested daily from Google, Bing and Baidu for four cities to reveal the relationship between dengue epidemics and the web search index. AIV H5 was detected in Dhaka city from week 38, peaking at week 39, while dengue virus was persistently detected from week 29 to week 45, aligning with clinical trends. Time-series cross-correlation analysis revealed that variations in surface water viral load led clinical case reports by approximately two weeks (max CCF = 0.572 at lag −2). In Ruili city, dengue virus was detected from week 32 to week 44. To sharpen sensitivity, 383 weekly web search series for 13 dengue keywords from four countries were screened; random-forest and XGBoost models retained five symptom queries that generated a composite index explaining 79% of variance in dengue RNA levels in an independent Ruili test set (*n* = 24) and reduced superfluous sampling by 35%. Phylogenetic analysis verified identity between water-derived and patient-derived DENV-2, confirming local transmission. The study demonstrates that AIV SWBS is optimally integrated with wild bird sampling for source attribution, whereas dengue SWBS achieves maximal efficiency when combined with real-time web search monitoring, providing tailored, low-cost early-warning modules for resource-constrained tropical settings.

## 1. Introduction

The transmission of zoonotic pathogens—such as avian influenza virus (AIV) and dengue virus (DENV)—from animals to humans has caused several devastating outbreaks in recent years [1]. In rural areas, public health authorities often fail to detect zoonotic infections at an early stage, particularly in remote regions with limited healthcare access and diagnostic capacity [2]. Consequently, these potentially epidemic- or pandemic-prone diseases often remain unreported, especially among populations with limited access to healthcare facilities. Farmed and wild animals in these regions are recognized reservoirs of these zoonotic pathogens [3].

Bangladesh and Yunnan Province in China, both situated in tropical to subtropical regions, face substantial threats from zoonotic diseases. Since 2007, Bangladesh has experienced multiple outbreaks of highly pathogenic AIV in poultry, with more than 550 outbreaks recorded and eight human cases of H5N1 reported, including one fatality [4]. In 2025, two human cases of H5N1 were reported in Khulna and Jessore districts of Bangladesh [5]. Concurrently, dengue fever has shown geographical expansion from core regions (Dhaka and Chattogram) to other areas [6]. This co-circulation of multiple pathogens underscores the significant challenges in Bangladesh and Yunnan province [7]. Yunnan Province is also confronted with imported cases of dengue fever from Myanmar and other regions [8]. Moreover, seasonal migration of wild birds further increases the risk of AIV introduction into Yunnan Province [9].

Wastewater-based surveillance (WBS) has recently emerged as a robust and essential tool for monitoring public health-relevant analytes by capturing pathogens shed by both symptomatic and asymptomatic individuals, irrespective of their socioeconomic status [10]. The U.S. Centers for Disease Control and Prevention (CDC) established a WBS covering 45% of the population to track the dynamics of AIV transmission. The system provides an early warning signal that both zoonotic and anthropogenic sources contribute to community infections [11]. During 2024 in the United States, wastewater monitoring across 48 states during an outbreak of highly pathogenic AIV (H5N1) consistently detected the AIV H5 subtype, confirming that wastewater monitoring serves as a One Health indicator for assessing influenza prevalence in communities [12]. Complementary research demonstrated the effectiveness of WBS by identifying H5 gene fragments coinciding with elevated prevalence of AIV in municipal wastewater solids in the spring of 2024 [13]. In addition, metagenomic sequencing of nine municipal wastewater samples was used to successfully identify H5N1 AIVs [14]. WBS has also demonstrated utility for early detection and assessment of the risk of dengue virus transmission in various countries [15,16,17]. However, rural areas frequently lack centralized sewer networks, posing challenges for implementing wastewater-based surveillance in these settings. Monitoring surface water in rural communities and wildlife habitats may offer a practical alternative [18]. Nevertheless, whether this approach can provide both early detection and comprehensive tracking of zoonotic outbreaks remains unclear.

To address this issue, in this study, we implemented surface water-based surveillance (SWBS) in a rural area of Bangladesh and China to detect AIV and dengue virus. Together with the sampling of wild animals or digital epidemiology, these findings not only validate the potential role of SWBS for early viral warning systems but also reveal the application value of a multifaceted approach for distinguishing the source of the virus.

## 2. Materials and Methods

### 2.1. Study Area and Surface Water Sampling

In this study, we implemented a Sewage and Water-Based Surveillance (SWBS) project in two locations: Dhaka, Bangladesh, and Ruili, China. Both locations face challenges due to limited resources for addressing zoonotic diseases and are situated within an intense monsoon climate regime, characterized by a distinct concentration of high-temperature rainy seasons [19,20]. This climatic setting provides favorable environmental conditions for the proliferation of vector mosquitoes and virus transmission. During the rainy season, inadequate drainage systems in Dhaka result in extensive surface water stagnation, forming persistent mosquito breeding habitats in public areas [21]. In contrast, Ruili’s mosquito proliferation primarily depends primarily on scattered water-holding containers at the household and community levels. These distinct hydrological conditions collectively create numerous microenvironments conducive to mosquito breeding. Furthermore, high-density urbanization and overburdened infrastructure not only increase human-vector contact frequency but also foster a high-risk environment for the cross-species transmission of pathogens such as avian influenza. Ruili’s position on the China-Myanmar border facilitates the continuous introduction of foreign pathogens through frequent cross-border human mobility and trade activities, including poultry trade [8,22]. Under suitable local climatic and hydrological conditions, these introduced pathogens can readily initiate local transmission cycles and potentially lead to exported cases.

In Bangladesh, we selected a remote suburban area of Dhaka (23.4813° N, 90.2040° E), which includes two sewage discharge outlets (Figure S1). The catchment area covers approximately 15 km^2^, with an average flow velocity of 0.5 m/s during the sampling period. The average monthly rainfall in Dhaka during sampling was 250 mm. From July to November 2023, surface water samples were collected weekly from 0.5 m below the surface at the designated sampling points. In China, river water was collected twice per week from the Ruili River in Ruili City (24.0125° N, 97.5149° E) (Figure S2). The catchment area is approximately 20 km^2^, with a relatively stable flow velocity of about 1.0 m/s. The average monthly rainfall in Ruili during the sampling period was 180 mm. To ensure consistency and reliability, the sampling frequency at both sites was standardized to twice per week. Surface water samples were collected using a Bag-Mediated Filtration System (BMFS), following the recommendations of a previous study [23]. A total of 3 L of water was filtered through a 0.22 μm pore-size polyethersulfone (PES) membrane (Millipore, Burlington, MA, USA). The filters were immediately stored at −20 °C prior to nucleic acid extraction using the QIAamp Viral RNA Mini Kit (Qiagen, Hilden, Germany).

### 2.2. Quantification and Subtyping of AIV in Surface Water

In this study, to monitor the presence of AIV in surface water, the M gene was first detected using RT-qPCR. A plasmid containing the AIV M gene (Sangon Biotech Co., Ltd., Shanghai, China) was used to generate a standard curve by performing a 10-fold serial dilution (10^1^–10^7^ copies/μL).

Subsequently, a multiplex one-step RT-qPCR assay was employed to identify five AIV subtypes (H1, H5, H7, H9, and H10). Primers and probes were designed using Primer Express 3.0 (Applied Biosystems, Foster City, CA, USA), and their sequences are listed in Table S1. Samples positive for the M gene but negative for all five HA subtypes were classified as AIV HA untyped.

To assess potential loss of viral genetic material during sample processing, pepper mild mottle virus (PMMoV) was used as a process control. PMMoV was propagated in *Escherichia coli* (ATCC 15597) cultures, harvested, and spiked into 35 mL of raw surface water at a concentration of 2.3 × 10^10^ PFU/mL. Recovery rates of PMMoV were determined by two methods: first, the modified PEG/precipitation method, followed by quantification of the active PMMoV using the double agar layer assay (in PFU/mL) as described by Cormier and Janes [24]; second, the PMMoV genome copies were quantified using RT-qPCR, following the method of Miranda and Steward [25]. Calibration curves were generated using dilutions of 1 × 10^0^, 1 × 10^1^, 1 × 10^2^, 1 × 10^3^, and 1 × 10^9^ PFU/mL (analyzed in duplicate). The recovery rate was calculated as the percentage of total inoculated viral particles recovered post-sample processing.

Quantitative detection was performed on the Applied Biosystems QuantStudio™ 3 Real-Time PCR System (Thermo Fisher Scientific, Shanghai, China). Each 20 μL reaction contained 10.0 μL Premix Ex Taq™ (Probe qPCR; TaKaRa, Shiga, Japan), 0.2 μm of each subtype-specific primer, 0.1 μm of the corresponding probe, 1.0 μL RNA template, and nuclease-free water to a final volume of 20 μL. The RT-qPCR program was as follows: reverse transcription at 50 °C for 15 min, initial denaturation at 95 °C for 2 min, followed by 40 cycles of 95 °C for 10 s and 58 °C for 30 s (annealing and extension). Each run included positive controls (standard plasmid) and negative controls (no template control). All tests were performed in triplicate.

### 2.3. Detection of Dengue Virus in Surface Water

For dengue virus (DENV), the capsid–premembrane (CprM) region of DENV was targeted for detection using RT-qPCR. A standard curve was established using tenfold serial dilutions (3 × 10^0^ – 3 × 10^5^ copies/μL) of CprM gene fragments.

Recovery efficiency for DENV was evaluated by spiking surface water samples with a known amount of DENV RNA and calculating the recovery percentage as (measured copies/spiked copies) × 100%. PMMoV was added as an internal amplification control in each reaction to monitor potential inhibition.

DENV detection was performed using the GoTaq^®^ 1-Step RT-qPCR System (Promega, Madison, WI, USA) following the method described by Johnson et al. [26]. Primers targeting the CprM region are shown in Table S1. Quantitative detection was performed on the Applied Biosystems QuantStudio™ 3 Real-Time PCR System (Thermo Fisher Scientific, Shanghai, China) in 20 μL reaction volumes. The thermal cycling conditions were: reverse transcription at 45 °C for 15 min, enzyme activation at 95 °C for 2 min, followed by 45 cycles of 95 °C for 15 s and 60 °C for 30 s. Each run included a positive control (standard plasmid) and a negative control (no-template control). All experiments were performed in triplicate.

### 2.4. Determination of LOD for AIV and DENV RT-qPCR Assays

The limits of detection (LOD) for the AIV and DENV RT-qPCR assays were determined using serial dilutions of pseudoviruses. For AIV, pseudoviruses with five different HA genes (Diff-BioTech, Shanghai, China), representing the M, H1, H5, H7, H9, and H10 genes, were used in serial dilution. For DENV, serial dilutions of DENV pseudoviruses (Zhongke Kaipu Biotechnology Co., Ltd., Wuhan, China) were employed.

Spiking experiments were performed by adding pseudoviruses at different concentrations (1 to 20 copies/μL) to AIV-negative or DENV-negative surface water samples, with 20 replicates for each concentration. The LOD was defined as the lowest concentration at which ≥95% of replicates tested positive. The 95% LOD was determined using probit analysis. Probit values were calculated using Microsoft Excel^®^ (Microsoft Corporation, Redmond, WA, USA) and plotted against the log_10_-transformed concentration data. The 95% LOD was then calculated from the regression equation corresponding to a probability of 0.95 (equivalent to a probit value of 1.64).

### 2.5. Collection of Fresh Bird Feces and RNA Extraction

Fresh fecal samples (*n* = 40) were collected weekly within a 3 km radius of positive sampling site (Figure S1). The feces originated from five wild bird species (Eurasian Hobby (*Falco subbuteo*), Oriental Magpie-Robin (*Copsychus saularis*), mallard duck (*Anas platyrhynchos*), Ruddy Shelduck (*Tadorna ferruginea*), Jungle crow (*Corvus levaillantii*)) and domestic chicken. Fresh feces from migratory birds were sampled from grass blades. The samples were collected early in the morning to minimize UV degradation. Fresh droppings were collected only when a single species was present, as confirmed by field surveys. For each sample, droppings from the same species were pooled to provide sufficient material, resulting in a final sample size of 1 g. The fecal samples were collected using a spoon and placed in a 15 mL sterile Falcon tube containing 2 mL of isotonic solution consisting of composed saline (PBS) with 50% glycerol, penicillin (10,000 U/mL), gentamicin (250 mg/mL), and nystatin (2500 U/mL). All samples were placed in sterile containers and transported to the laboratory in Chattogram Veterinary and Animal Sciences University on ice within 24 h of collection. Viral RNA was extracted from bird feces using QIAamp^®^ Viral RNA (QIAGEN, Hilden, Germany) according to the manufacturer’s instructions. The presence and subtyping of the AIV was conducted by RT-qPCR as detailed in above section.

### 2.6. Sequencing of Eight Fragments for AIV

Amplification of the eight segments of AIV was carried out by standard conventional RT-qPCR, as described previously. Among the RT-qPCR positives, the samples with Ct value ≤33 were considered for sequencing. Library preparation was performed using the MGISP-100RS automated sample preparation system (CAT No. 900-000070-00). Sequencing was conducted on the DNBSEQ-E25 sequencer using the DNBSEQ-E25RS high-throughput sequencing reagent kit (FCL PE150) (MGI Tech Co., Ltd., Shenzhen, China). The sequencing data were aligned to the AIV H5N6 reference genome (A/Whooper swan/Mongolia/25/2020) using the MGI FluTrack software (version v1.0).

### 2.7. Phylogenetic Analysis of AIV Fragments

The obtained sequences were subjected to Clustal W multiple sequence alignment and residue analyses using the BioEdit 7.1.5 program. HA subtypes and nucleotide identity were confirmed using basic local alignment (BLASTn) searches (https://blast.ncbi.nlm.nih.gov/Blast.cgi, accessed on 26 October 2025). Clade information was identified using the H5 clade classification tool incorporated within the Influenza Research Database. We downloaded (2 June 2024) all HA sequences of H5 viruses (*n* = 564) deposited in the Global Initiative for Sharing All Influenza Data (GISAID; https://gisaid.org/) [27] since 2010. Sequences shorter than 1500 bp or lacking complete metadata were excluded. We only kept sequences from chicken, duck, and wild birds, resulting in a total of 429 sequences.

Sequences were aligned using the MAFFT [28]. The best fit substitution model was identified as the lowest BIC using ModelFinder within IQ-TREE [29], and we inferred maximum likelihood phylogenetic trees, using IQ-TREE version 1.68, with 1000 bootstraps. In addition to these sequences from Bangladesh, we used the BLASTn search tool to identify closely related sequences belonging to clade 2.3.4.4 in both GenBank and the GISAID Epiflu database. Similarly, NA, M, NS, and NP sequences from Bangladesh and China were also downloaded from GISAID. Phylogenetic relationships of above genes were also analyzed as described above.

### 2.8. Sequencing of Dengue Virus from Surface Water

The CprM gene in the viral polyprotein gene was amplified by standard conventional RT-qPCR, which was performed as previously described by Gomes et al. [30]. Of the RT-qPCR-positive samples, those with Ct values ≤ 33 were selected for sequencing. Phylogenetic relationships of CprM gene were analyzed using the same approach employed for AIV.

### 2.9. Collection of Clinical Data

We collected dengue fever case data from Bangladesh, provided by the World Health Organization [31]. All reported dengue cases from Ruili City in 2023 and Guangzhou or Shenzhen in 2024 were obtained through the local Disease Control and Prevention or literature [32]. Statistical analyses were performed using the R package v4.3.2, Pearson’s correlation analysis was employed to evaluate the association between the number of infected patients and abundance of dengue virus in surface water.

To quantitatively assess the temporal lead-lag relationship between environmental surveillance data and clinical case reports, a cross-correlation function (CCF) analysis was conducted. This method computes correlation coefficients across a range of time lags, identifying the specific lag at which the two time series exhibit the strongest association. Prior to CCF analysis, a prewhitening procedure was applied to both time series to remove internal autocorrelation, thus preventing spurious correlations. All analyses were performed using the ‘forecast’ package in R software (version 4.3.2). For a time series of length *n*, the 95% confidence interval for the cross-correlation coefficients was defined as $\pm \frac{1.96}{\sqrt{n}}$ . A correlation peak exceeding this threshold was considered statistically significant.

### 2.10. Derivation of Web Search Sentiment Indices for Dengue

Web search activity was used as a proxy for public sentiment towards dengue. Indices were extracted from Google Trends (https://trends.google.gg), the Bing Webmaster Index (https://www.bing.com/webmasters) and the Baidu Index (http://index.baidu.com) for 1 February 2022–31 July 2023. Next, we adopted a three-step approach for understanding the role of web search activity for the early warning of dengue.

Step 1—Keyword compilation

The term “dengue” was queried weekly on each platform. All 50 unique indices returned were pooled, and their weekly search volumes were averaged across platforms. The 15 indices with the highest mean volume were retained for further analysis.

Step 2—Daily search volume acquisition

Weekly indices were collected for Dhaka (Bangladesh) and Ruili (China) concomitantly with wastewater sampling. Analogous weekly data were obtained from two prior European studies (Portugal, *n* = 273; Italy, *n* = 30) covering both mobile and PC traffic [16,33]. Linear regression between the 13 candidate indices and the corresponding wastewater dengue RNA concentrations yielded five indices with R^2^ > 0.70 (*p* < 0.05). Daily average search volumes for these five indices were extracted for the same cities and periods.

Step 3—Machine learning modelling

Three algorithms—linear regression (LM), random forest (RF) and extreme gradient boosting (XGB)—were trained to predict dengue virus RNA levels in wastewater from the 13 retained search terms (Table S3). Training data comprised 280 samples: Portugal (*n* = 273), Italy (*n* = 30), China (*n* = 40) and Bangladesh (*n* = 40). LM was implemented with the lm function (R 4.3.2, stats package). For RF, the percentage increase in mean squared error (%IncMSE) was calculated over 1000 permutations; the relative importance of each term was expressed as the %IncMSE of the term divided by the total %IncMSE of all terms. For XGB, mean importance (IMP) was computed from the same 1000 permutations. Terms were retained if they met any of the following criteria: R^2^ ≥ 0.70 in LM; contribution ≥ 10% in RF; or IMP ≥ 10% in XGB.

Model performance was evaluated on an independent test set of 24 samples collected in Ruili city (1 July–15 September 2024) and validated on another 24 samples from Ruili city in the next two months (16 September–7 December 2024) (Figure S2). Wastewater sampling, RNA extraction and RT-qPCR were performed as described previously.

## 3. Results

### 3.1. Performance of RT-qPCR Assays for AIV and DENV Detection

The standard curves for AIV and DENV assays demonstrated high linearity and amplification efficiency (Table S2). For AIV, the M gene and five HA subtypes (H1, H5, H7, H9, and H10) all exhibited strong correlations (R^2^ = 0.991–0.999) and amplification efficiencies ranging from 97.6% to 99.8%, indicating excellent assay performance (Figure S3). The LOD for all AIV targets ranged from 1.1 to 1.2 log_10_ copies per reaction. The DENV assay targeting the CprM gene also showed reliable quantification, with an R^2^ value of 0.985, amplification efficiency of 99.8%, and an LOD of 1.5 log_10_ copies per reaction (Figure S4). These results demonstrate that both RT-qPCR systems were highly sensitive and reproducible for the quantitative detection of AIV and DENV RNA in surface water samples.

### 3.2. Detection of AIV in Surface Water

**Figure 1:**
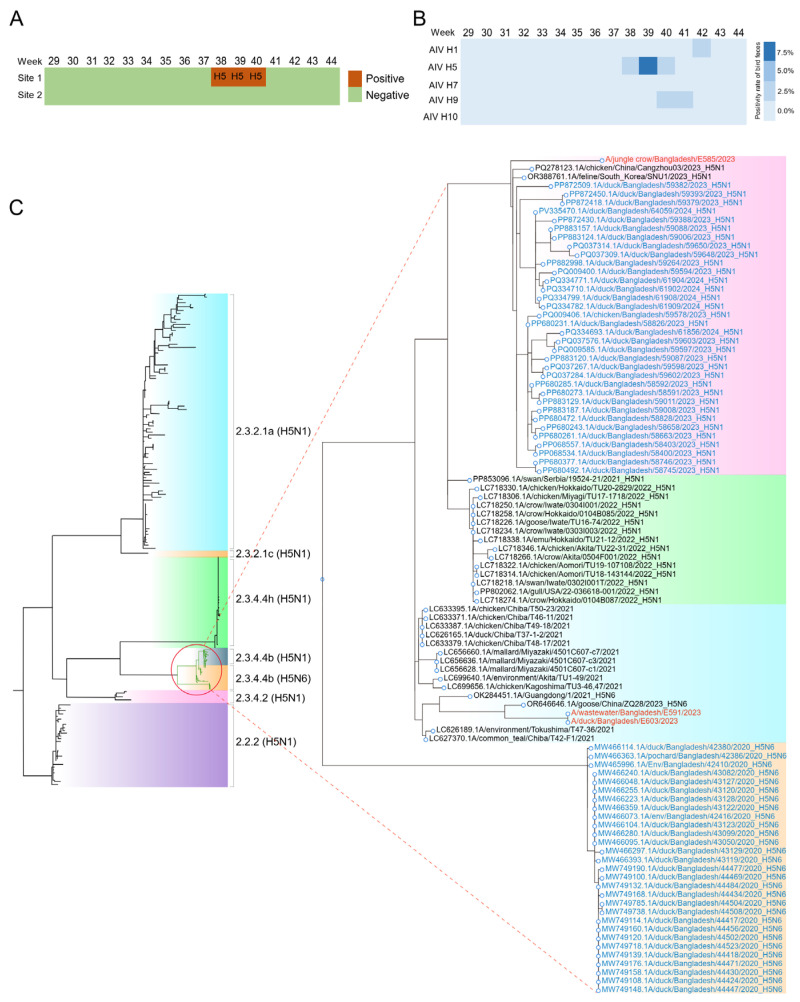
The presence and phylodynamic analysis of AIV in surface water and fecal samples. (**A**) The presence of AIV in two sampling sites of Dhaka city from July to November 2023; (**B**) The presence of AIV in fecal samples from wild birds from week 29 to 44, 2023; (**C**) Phylogenetic relationship of the HA genes of the H5Nx viruses between surface water and wild birds in South Asia. The viruses identified in this study are shown in color. Bootstrap values ≥ 70 are shown on branches.

For Dhaka city, AIV H5 was detected at site 1 from weeks 38 to 40, with concentrations of 1121, 23,500, and 3625 copies/L, respectively, indicating a clear peak in viral load during week 39 (Figure 1A). Meanwhile, AIV H1 and H9 were found in site 1 in weeks 40–41 and 42, respectively. AIV H1, H5, H7, H9, and H10 were negative for site 2 during the sampling period. Among these samples, only one sample had a Ct value below 33 for H5. Sequencing was performed for the above samples and confirmed H5N6 was presented in the surface water. AIV was not detected in the Ruili River during the study period.

### 3.3. Identification of AIV in Bird Feces

As there is no human infection of H5N6 reported by public health department of Bangladesh during the study period, to assess the origin of AIV found in surface water, we conducted weekly sampling of wild bird and domestic chicken feces at a nearby wildlife sanctuary. Sequencing data quality metrics are summarized in Table S3. Results showed that AIV-positive samples for H1 (*n* = 1), H9 (*n* = 3), and H5 (*n* = 5) subtypes (Figure 1B). Three H5-positive and one H1- and H9-positive samples with low Ct values were used for sequencing, which subtyped them into H5N6, H1N3, and H9N2, respectively. However, only NS and M were sequenced with over 80% completeness for all samples (Table S4). In addition, HA, NA gene coverage ranged from 60.8 to 85% and PB2 gene coverage ranged from 60.8 to 74.55%. This non-uniform coverage profile limited the phylogenetic resolution for certain genomic segments. HA, NP and NA were identified in three samples.

### 3.4. Phylogenetic Analysis of HA and NA of AIV Subclades

To determine the evolutionary relationships between AIVs detected in surface water and wild bird feces, we performed phylogenetic reconstruction of five genomic segments (HA, NA, M, NS, NP) using maximum likelihood methods. Phylogenetic analysis of the HA gene from H5Nx viruses collected across East and South Asia revealed that two HA sequences from wild birds clustered with one from surface water. These sequences were most closely related to previously reported H5N6 viruses detected in China in 2023 (Figure 1C). Another bird-derived HA sequence exhibited greater genetic relatedness to a virus isolated in Hebei Province. The observed genetic relatedness suggests limited sequence divergence among regional H5Nx strains.

The maximum likelihood (ML) tree, constructed using representative NA sequences from Bangladesh and East Asia, suggested that all NA genes sequenced in this study likely originated from an H5N6 ancestor in wild birds from Guangdong, China, in September 2023. This ancestor was located within a branch comprising H5N6 virus isolates (Figure S5). Intriguingly, the NP genes showed varying relatedness to reference sequences from Bangladesh and neighboring regions, suggesting genetic diversity among the detected strains (Figures S6–S8).

For H1N3 viruses identified in wild birds, phylogenetic analyses of NA genes suggested that both likely originated from China (Figure S9), highlighting the potential for long-distance transmission of AIVs.

### 3.5. Detection of Dengue Virus in Surface Water from Dhaka City

**Figure 2:**
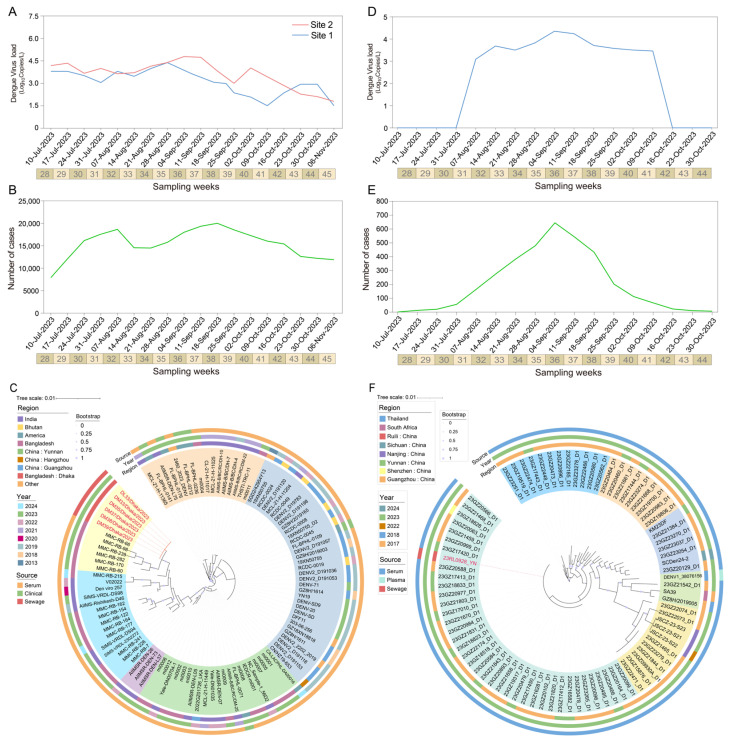
Spatio-temporal dynamics and phylogenetic analysis of dengue viruses from surface water monitoring. (**A**) Viral RNA concentration (log_10_ copies/L) of CprM gene in two surface water samples from Dhaka, Bangladesh, detected by RT-qPCR; (**B**) Dhaka corresponds to the number of clinically confirmed dengue cases per week; (**C**) Maximum likelihood phylogenetic tree of CprM gene sequences in Bangladesh; (**D**) Dengue virus RNA concentration in surface water samples from Ruili River, China; (**E**) Corresponding number of clinical cases in Ruili city; (**F**) Phylogenetic analysis of CprM sequences in Ruili. Viruses identified in this study are labeled in color. Bootstrap values shown are ≥70. All sequences were categorized into DENV-1 serotypes. Scale bars indicate the number of nucleotide substitutions per site.

In this study, we investigated the temporal variations in dengue virus concentrations in surface water samples. These samples collected from two sites in Dhaka city between July and November 2023 (Figure 2A). Over the entire study period, site 2 exhibited consistently higher mean viral concentrations compared to site 1, peaking at 4.62 log10 copies/L during weeks 36–37. After week 41, the concentration of dengue virus at site 1 began to rise, eventually surpassing that of site 2 by week 43.

During epidemiological weeks 32 to 35 (28 August to 15 September 2023), the concentration of dengue virus increased synchronously at both sampling sites. Meanwhile, a surge of 5524 new clinical cases reported during weeks 35 to 38 (15 September to 1 October 2023) (Figure 2A). A significant positive correlation (r = 0.572) at lag −2 weeks was found by cross-correlation analysis, suggesting that changes in surface water virus concentrations preceded clinical case reports by approximately two weeks (Figure S10).

A total of six surface water samples were successfully sequenced for the CprM gene. Phylogenetic analysis showed that the six samples from this study (DL33, DM33, DM35, DM37, DM38, DM39) clustered closely with the dengue virus type 2 isolate MMC-RB-66 derived from a serum sample in Dhaka (GenBank: PP704405.1) (Figure 2C). Clinical reported showed that DENV2 re-emerged as the main circulating serotype during the 2023 outbreak, with the overall proportion of circulating serotypes being DENV-2 (68.1%), DENV-3 (25.4%), DENV-1 (2.2%), DENV-4 (0.2%), and mixed infections [31]. This finding is consistent with the sequencing results from clinical samples, suggesting the DENV-2 was main genotype circulating during this period (Table S5).

### 3.6. Detection of Dengue Virus in Surface Water from Ruili City

SWBS along the Ruili River in the border region of China revealed sustained high concentrations of dengue virus from August to November 2023, ranging from 3.2 to 4.5 log10 copies/L and peaking in week 36 (early September) (Figure 2C).

During 2023, a total of 3824 autochthonous dengue cases were reported in Ruili City, Yunnan Province, China [32]. Case numbers began to rise in July, peaked in September (*n* = 2391), and gradually declined in October (Figure 2E). The temporal trends of viral loads in surface water were strongly correlated with the number of clinical cases (Pearson’s r = 0.934, *p* < 0.001) (Figure S11).

Phylogenetic analysis revealed that the sequences obtained in this study clustered with a group of strains isolated from serum samples in Yunnan and Guangzhou during 2023, all of which belong to the DENV-1 serotype. The Nanjing variant was found to be closely related to the Guangzhou 23GZ22073_D1 strain (GenBank: PP563947.1). Additionally, international strains SA39 and DENV1_38076156 formed a distinct clade with a shared ancestor, suggesting potential inter-regional transmission between Yunnan, Guangzhou, Nanjing, and Southeast Asia (Figure 2F, Table S5).

### 3.7. Integration of Digital Epidemiology with SWBS

**Figure 3:**
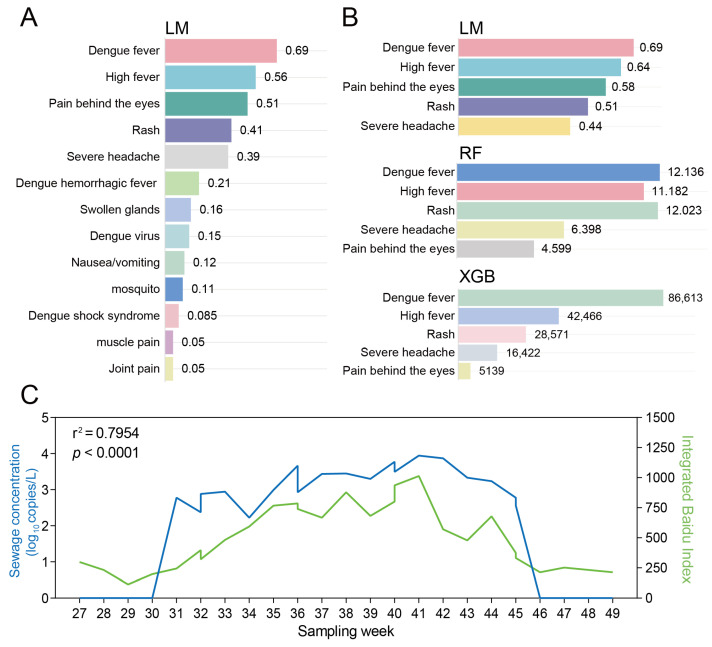
Correlation analysis between dengue virus concentration in wastewater and web search indices in Ruili, China. (**A**) Correlation between a collection of 13 web search indices and sewage concentration; (**B**) Selection of the top 5 web search indices most correlated with the sewage concentration of dengue virus in wastewater, based on LM, RF (measured by %IncMSE), and XGB models (measured by IMP); (**C**) Validation the correlation between dengue virus concentration in wastewater and web search indices in Ruili, China in 2024.

To enhance the monitoring efficiency of dengue fever, we selected 50 dengue fever—related web—search terms and analyzed the relationship between their search volumes and sewage concentrations. Of the 50 dengue-related keywords monitored, 13 were consistently ranked among the top queries on Google, Bing and Baidu (Table S6). Weekly wastewater samples collected in Dhaka, Ruili, Porto and Rome were paired with the corresponding local search indices for these 13 terms. Simple linear regression revealed strong associations between the two data streams; the five best-performing symptom-related terms—“Dengue fever”, “Rash”, “Severe headache”, “High fever” and “Pain behind the eyes”—yielded R^2^ > 0.469 (Table S7, Figure 3A).

To refine the signal, RF, XGB and LM models were trained on the wastewater concentrations from the four cities and the daily search volumes of the top five terms. All three algorithms converged on the same five keywords as the most predictive features (Figure 3B, Tables S8 and S9). Aggregating these terms into a composite index produced an even stronger correlation with viral RNA levels (R^2^ = 0.795, *p* < 0.01) when validated against 18 weekly samples from Ruili (July–November 2024) (Figure 3C). Critically, dengue virus was only detected in wastewater when the composite index exceeded a predefined threshold, indicating that sampling frequency can be dynamically adjusted to online search trends.

## 4. Discussion

In this study, we conducted SWBS in a rural area near Dhaka city, Bangladesh and Ruili city, China from July to November 2023 to evaluate the early-warning capacity of SWBS for AIV and dengue virus. The results showed that continuous detection of both AIV and dengue viruses in the surface water in a rural area of Dhaka is feasible. After the detection of AIV H5N6 in sewage, we compared our data with those from public health authorities and found no evidence of human cases of avian influenza during this period. A report from the United States also documented a similar phenomenon, with AIV detected in sewage, but no corresponding cases identified in the human population [12]. It was speculated that the AIV in sewage might originate from poultry. To investigate this, we collected fecal samples from wild birds and poultry around the sewage sampling sites and tested them using RT-qPCR. We identified multiple avian influenza genotypes, including H5N6, H9N2, and H1N3 in wild birds. Sequencing analysis revealed that the HA and NA genes of H5N6 in sewage were homologous to those in wild birds, suggesting that wild birds may be one source of AIV in sewage. Nevertheless, we did not rule out the possibility of the contribution of poultry. Live poultry markets in Bangladesh are high-risk sites for AIV transmission [34]. Results herein suggested that SWBS can promptly detect the presence and spread of AIV, thereby implementing effective preventive measures to reduce the risk of virus transmission to humans and wild birds.

In recent years, dengue fever has also been a severe public health issue in Bangladesh and Yunnan Province of China. A previous study suggested that wastewater surveillance at WWTPs or community can act as a powerful tool to provide near real-time monitoring results for the entire population in a cost-effective manner [35,36]. However, the detection of dengue fever in surface water has not yet been reported. Recently, Gouthro et al. (2025) demonstrated the potential of surface water surveillance using passive samplers as a scalable strategy for detecting AIVs with successful detection of AIVs and hemagglutinin subtype H5 genes in surface waters across various sites in Nova Scotia, Canada [37]. However, it is still uncertain whether this method can track the entire epidemic cycle of dengue fever. In this study, SWBS for DENV effectively tracks the epidemic cycle of dengue, with viral concentrations in wastewater preceding clinical case reports by approximately two weeks and correlating significantly with case counts (r = 0.934, *p* < 0.001), thus providing a robust early warning signal for dengue outbreaks.

In the surveillance of dengue fever, given the scarcity of public health personnel in rural areas, which makes long-term and sustained mosquito density monitoring difficult, we established an early warning framework for dengue fever in rural regions by integrating SWBS with web searches related to dengue fever symptoms. While recent studies have also employed web search engine data to predict dengue fever outbreaks, the current search terms are often limited to the word “dengue” itself [38]. In this study, we further expanded the search scope to include all symptoms related to dengue fever and ranked these search keywords according to their relevance using machine learning algorithms. This approach is expected to significantly enhance the efficiency of detecting early dengue fever cases in rural areas and would effectively compensate for the delay in dengue fever early warning caused by the lag in clinical monitoring in rural areas. The five symptom-related queries captured >80% of the predictive power, suggesting that syndromic keywords alone are sufficient for operational surveillance. Nevertheless, search data cannot discriminate dengue from other arboviral infections that provoke similar symptoms. Thus, we suggested that wastewater surveillance together with real-time web search data can complement each other’s strengths and weaknesses and create a two-tier early-warning system. By initiating enhanced wastewater sampling only when web search indices for dengue symptoms exceed a predefined threshold, the frequency of sampling can be dynamically adjusted. This approach not only maintains the efficiency of detecting viral circulation but also minimizes unnecessary sampling efforts, thereby lowering the overall cost of surveillance while ensuring timely public health responses. This workflow was successfully piloted in Ruili during 2024; no additional field resources were required beyond routine sewage collection. The integration of digital epidemiology with environmental surveillance is particularly suited to resource-limited rural settings where traditional syndromic reporting is sparse. By using freely accessible search-engine data to modulate wastewater sampling intensity, public-health authorities can allocate testing kits, vector-control teams and clinical supplies more efficiently and at lower cost.

This study is also subject to a few limitations. First of all, environmental conditions, particularly rainfall and water flow, can significantly influence viral detection and persistence in surface water. Heavy rainfall may dilute viral concentrations, reducing the likelihood of detection, while reduced water flow can concentrate viruses, enhancing detection rates. In our study, we collected samples during the period from July to November 2023, which corresponds to the rainy season in Bangladesh and China. This period may have influenced our detection rates. Future studies should consider longer-term sampling to better account for seasonal variations and their impact on viral detection. Secondly, the relatively short surveillance window (July–November 2023) limits the ability to generalize temporal patterns or assess inter-annual variability. While our study demonstrates the feasibility of WBS for detecting zoonotic viruses in rural settings, longer-term studies are necessary to fully understand the dynamics of these viruses. Inter-annual studies would be particularly valuable to capture variability in viral prevalence and to better inform public health strategies.

Overall, we present an integrated surveillance framework that combines SWBS, wild bird monitoring, and web-based search data to enhance early detection and response to AIV and dengue virus in rural settings. Our findings demonstrate that SWBS can effectively capture the temporal dynamics of two virus cycles, significantly improving the timeliness and coverage of surveillance compared to traditional methods. By detecting AIV in surface water, we can strategically guide wild bird sampling efforts based on viral concentration and geographic distribution of positive samples. This targeted approach reduces sampling effort and cost while enhancing surveillance efficiency. Furthermore, integrating SWBS with web search data shows promise for improving early detection of dengue fever cases in rural areas, where clinical reporting is often delayed. This combination helps mitigate the lag in traditional surveillance systems and enables more timely public health responses.

In summary, this study effectively addressed its primary objectives. First, we established the feasibility of SWBS by successfully tracking the transmission dynamics of both avian influenza (AIV) and dengue viruses in the resource-limited rural settings of Bangladesh and China. Second, we validated its correlation with clinical data, demonstrating a significant lead time of approximately two weeks for dengue and confirming the identity of water-derived and clinical viruses through genomic sequencing. Finally, by integrating SWBS with wild bird sampling and web search indices, we developed a cost-effective, scalable, digital-environmental integrated surveillance framework.

## 5. Conclusions

To the best of our knowledge, this study is the first to document the detection of dengue virus RNA in rural tropical surface-water habitats and to integrate these data with real-time, machine learning-enhanced web search signals. By coupling SWBS with digital epidemiology in peri-urban Dhaka and Ruili, we delivered a low-cost, scalable early-warning system that pre-dated clinical alerts by two weeks and explained up to 79% of variance in viral loads. The concurrent recovery of H5N6 and DENV-2 genomes from water that matched avian and patient sequences further validates SWBS as a One-Health tool for resource-limited settings, which holds promise as a valuable tool for the early warning of zoonotic diseases in these regions.

## Data Availability

Raw sequencing data of AIV were deposited in GenBank (NCBI) under Accession No. PV622343, PV627868-PV635187, PV636433-PV635136, PV636466-67, PV636479-80, PV636490, PV636494.

## Supplementary Materials

The following supporting information can be downloaded at: https://www.mdpi.com/article/10.3390/microorganisms13112526/s1, Table S1. Real-time PCR primers and probes of two viruses; Table S2. Standard curve of M, H1, H5, H7, H9, and H10 gene of avian influenza viruses (AIV) and CrpM gene of dengue virus (DENV); Table S3. Summary of sequencing data quality metrics; Table S4. General information of sequence data of AIV; Table S5. Summary of Virus Subtypes Identified in Surface Water-Based Surveillance (SWBS) and Clinical Cases, and Their Phylogenetic Relatedness; Table S6. Correlation with wastewater concentrations of dengue virus within 13 Baidu indexes; Table S7. Using LM model to screen Baidu index related to wastewater concentration; Table S8. Using RF model to screen Baidu index related to wastewater concentration; Table S9. Using XGB model to screen Baidu index related to wastewater concentration; Figure S1. Two sampling sites in Dhaka city of Bangladesh from July to November 2023. Site 1 and Site 2 are indicated by red points. The catchments of Site 1 and Site 2 are indicated by blue and orange dotted lines, respectively; Figure S2. Sampling site in Ruili city of Yunnan Province, China from July to November 2023. Ruili is a county-level city located in the western part of Dehong Prefecture, Yunnan Province, China, bordered by Myanmar to the east, south, and west. The sampling site is situated along the Ruili River, 8 km southeast of Ruili city. Panels: A, location of Yunnan Province; B, location of Ruili city; C, sampling site in Ruili city (red point); Figure S3. Standard curves of viruses were used for the determination of the sewage concentrations. (A) Standard curve of M gene of AIV; (B) Standard curve of H1gene of AIV; (C) Standard curve of H5 gene of AIV; (D) Standard curve of H7 gene of AIV; (E) Standard curve of H9 gene of AIV; (F) Standard curve of AIV-H10; (G) Standard curve of dengue virus; Figure S4. RT-qPCR detection limits for M (A), H1 (B), H5 (C), H7 (D), H9 (E), and H10 (F) genes of avian influenza virus viruses and CrpM gene of dengue virus. 95% confidence limits of detection (LOD) for RT-qPCR assays were estimated using logistic regression models; Figure S5. Phylogenetic relationship of the NA genes of the H5N6 viruses isolated from wild bird feces in Dhaka. Maximum-likelihood tree inferred with IQ-TREE v1.68 (1000 bootstraps). Viruses identified in this study are shown in red. Bootstrap values ≥ 70 are shown on branches; Figure S6. Phylogenetic relationship of the NP genes of the H5N6 viruses isolated from wild bird feces in Dhaka. Maximum-likelihood tree inferred with IQ-TREE v1.68 (1000 bootstraps). Viruses identified in this study are shown in red; viruses from Bangladesh are marked in green; viruses from South Korea are marked in blue. Bootstrap values ≥ 70 are shown on branches; Figure S7. Phylogenetic relationship of the M genes of the H5N6 viruses isolated from wild bird feces in Dhaka. Maximum-likelihood tree inferred with IQ-TREE v1.68 (1000 bootstraps). Viruses identified in this study are shown in red; viruses from Bangladesh are marked in green; viruses from Komsomolsk-na-Amure are marked in blue; viruses from South Korea are marked in purple. Bootstrap values ≥ 70 are shown on branches; Figure S8. Phylogenetic relationship of the NS genes of the H5N6 viruses isolated from wild bird feces in Dhaka. Maximum-likelihood tree inferred with IQ-TREE v1.68 (1000 bootstraps). Viruses identified in this study are shown in red. Bootstrap values ≥ 70 are shown on branches; Figure S9. Phylogenetic relationship of the NA genes of the H1N3 viruses isolated from wild bird feces in Dhaka. Maximum-likelihood tree inferred with IQ-TREE v1.68 (1000 bootstraps). Influenza A viruses from this study are marked in red; viruses from Jiangxi, China are marked in blue; viruses from Zhejiang, China are marked in green. Bootstrap values ≥ 70 are shown on branches; Figure S10. Temporal relationship between surface water dengue virus detection and clinical surveillance in Dhaka, Bangladesh. (A) Time series of average dengue virus RNA concentration in surface water (blue line, left y-axis) and clinically reported dengue cases (red line, right y-axis) from epidemiological weeks 28–45, 2023. (B) Cross-correlation function (CCF) analysis between surface water viral load and clinical cases. The dashed red lines indicate the 95% confidence interval (±0.462); Figure S11. Regression analysis between DENV load in surface water and clinical cases in Ruili City. A significant linear relationship was observed between dengue virus load in the Ruili River and the number of weekly clinical cases. The linear regression equation is y = 0.0489x + 3.3492, with a coefficient of determination (r^2^) of 0.8724.

## Abbreviations

The following abbreviations are used in this manuscript:

SWBS	Surface water-based surveillance
DENV	Dengue virus
AIV	Avian influenza virus
