# Supplementary material for: Harnessing a Surface Water-Based Multifaceted Approach to Combat Zoonotic Viruses: A Rural Perspective from Bangladesh and China
Source: Microorganisms. 2025 Nov 4;13(11):2526. doi: 10.3390/microorganisms13112526 (PMC12654024; doi:10.3390/microorganisms13112526)
Supplement: Supplementary file 1 [file microorganisms-13-02526-s001.zip › microorganisms-3848244-supplementary.pdf]

Supplementary Figure

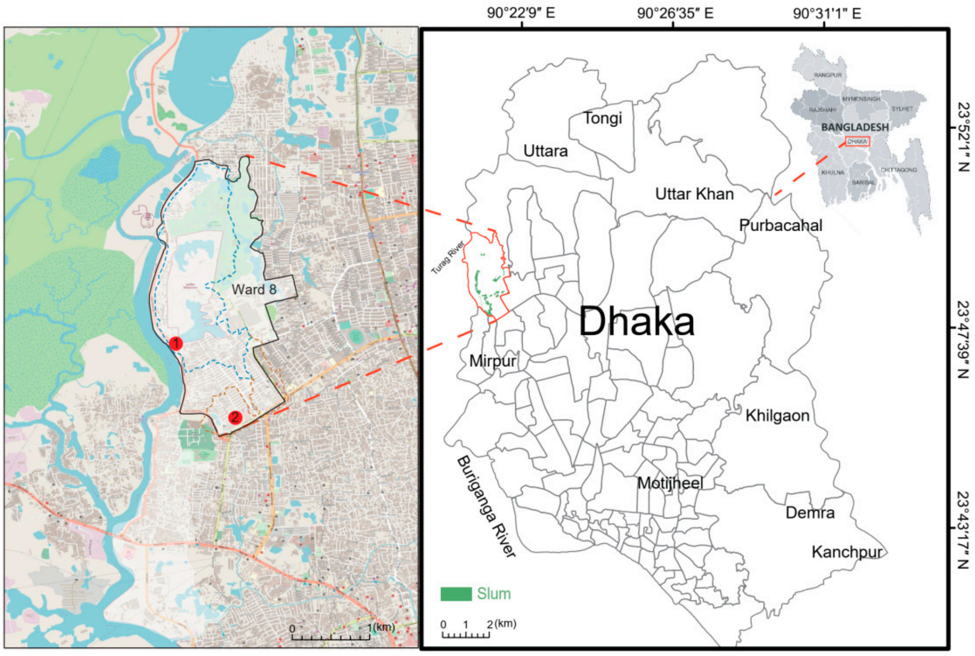

Figure S1 Two sampling sites in Dhaka city of Bangladesh from July to November, 2023. Site 1 and site 2 are indicated in red points. The catchment of site 1 and 2 are indicated in blue and orange dot lines, respectively.

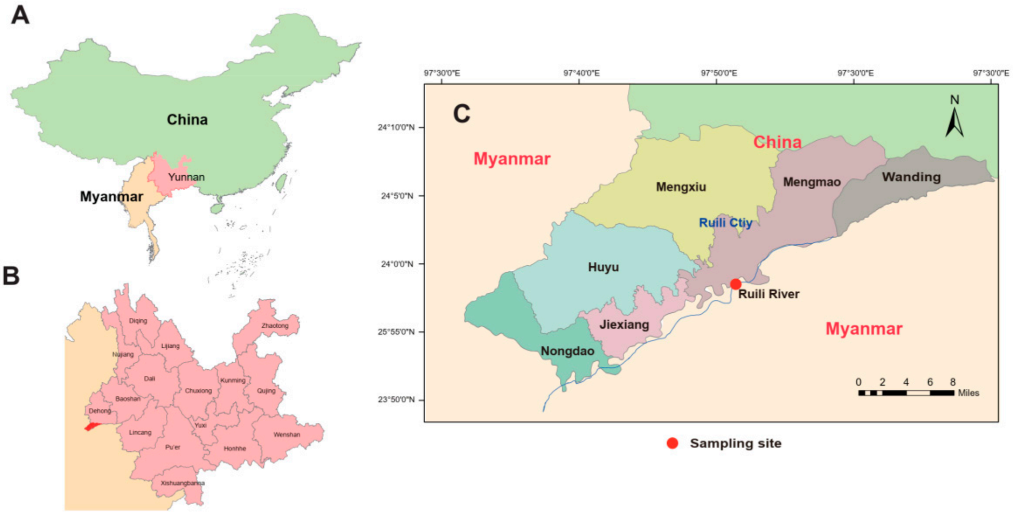

Figure S2 Sampling site in Ruili city of Yunnan Province, China from July to November, 2023. Ruili is a county-level city located in the western part of Dehong Prefecture, Yunnan Province, China. It is bordered by Myanmar to the east, south, and west. The sampling site is situated along the Ruili River, 8 km southeast of Ruili city. A: location of Yunan province; B: location of Ruiling city; C: Sampling site in Ruiling city. Sampling site is indicated in red point.

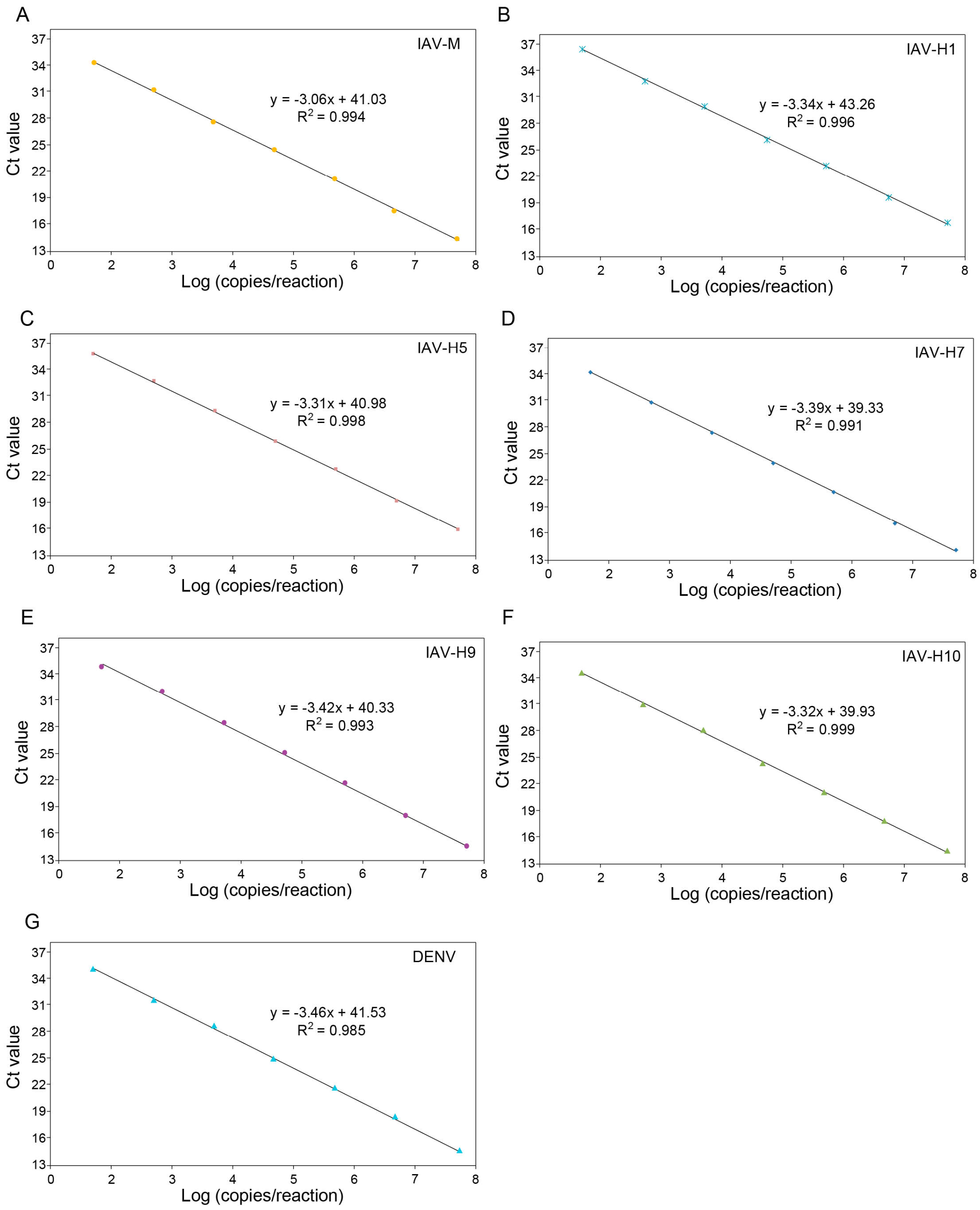

Figure S3 Standard curves of viruses used for the determination of the sewage concentrations. A: Standard curve of M gene of AIV; B: Standard curve of H1gene of AIV; C: Standard curve of H5 gene of AIV; D: Standard curve of H7 gene of AIV; E: Standard curve of H9 gene of AIV; F: Standard curve of AIV-H10; G: Standard curve of dengue virus.

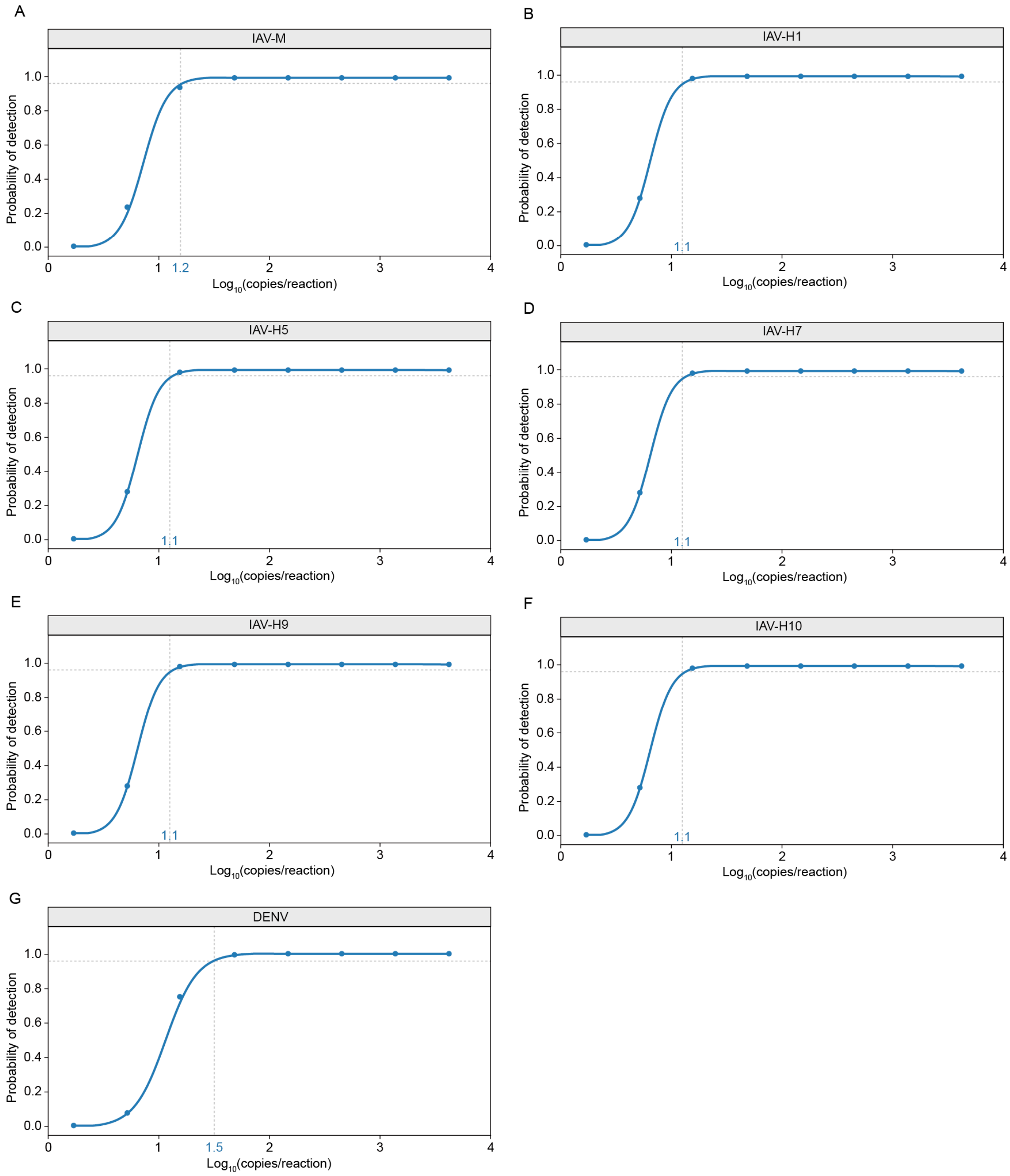

Figure S4 RT-qPCR detection limits for M (A), H1(B), H5(C), H7(D), H9(E), and H10(F) gene of avian influenza virus viruses and CrpM gene of dengue virus (G). 95% confidence limits of detection (LOD) for RT-qPCR assays were estimated using logistic regression models.

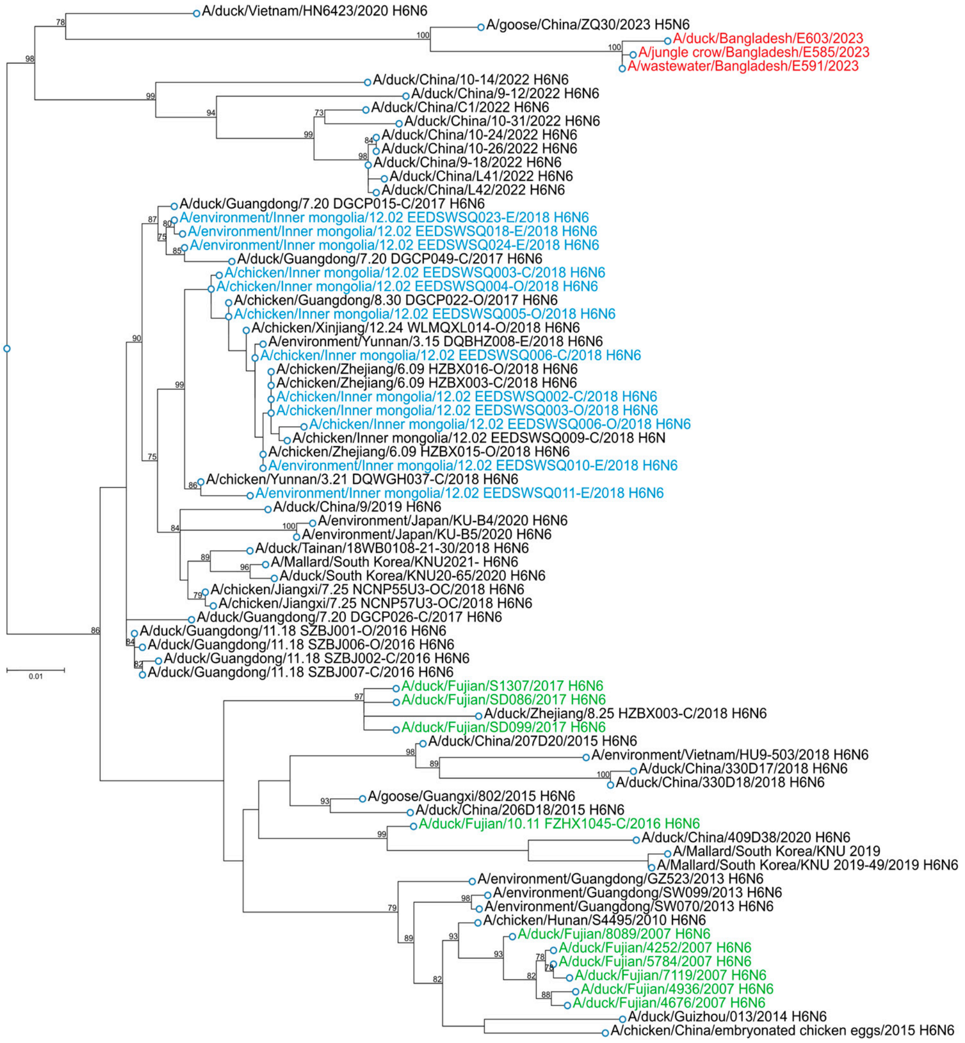

Figure S5 Phylogenetic relationship of the NA genes of the H5N6 viruses isolated from the wild bird feces in Dhaka. The maximum likelihood phylogenetic tree was inferred, using IQ-TREE version 1.68, with 1000 bootstraps. The viruses identified in this study are shown in red. Bootstrap values ≥70 are shown on branches.

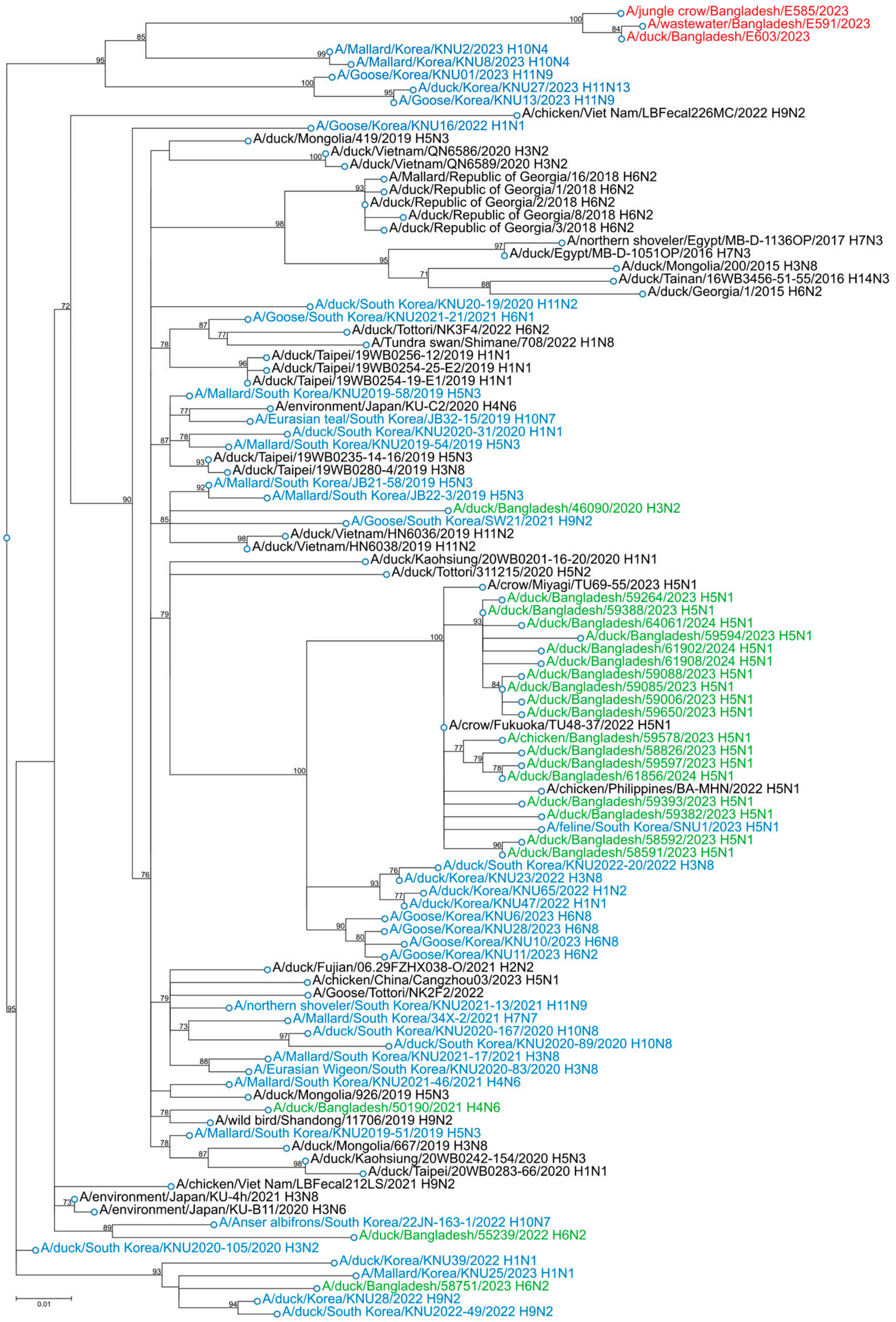

Figure S6 Phylogenetic relationship of the NP genes of the H5N6 viruses isolated from the wild bird feces in Dhaka. The maximum likelihood phylogenetic tree was inferred, using IQ-TREE version 1.68, with 1000 bootstraps. The viruses identified in this study are shown in red. viruses from Bangladesh are marked in green; viruses from South Korea are marked in blue. Bootstrap values ≥70 are shown on branches.

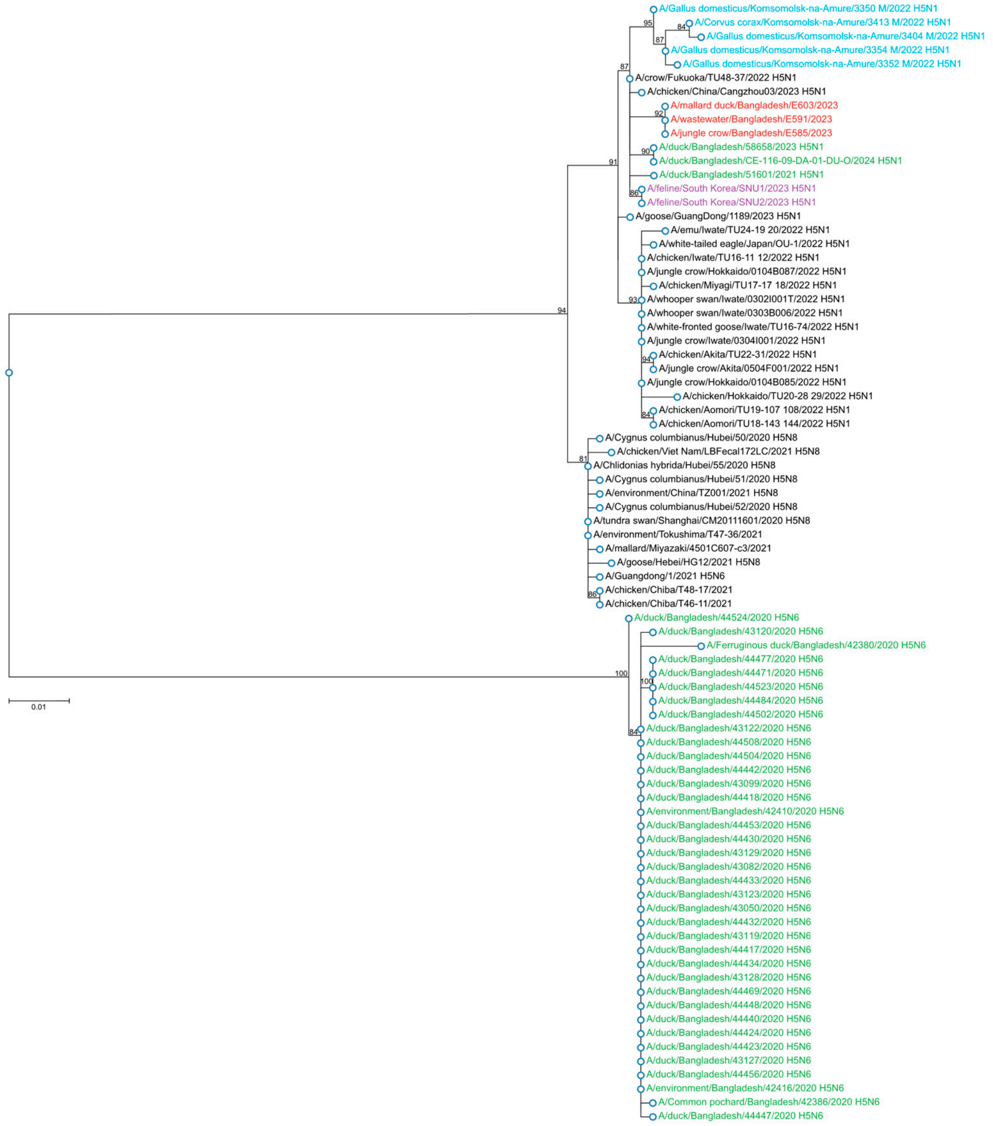

Figure S7 Phylogenetic relationship of the M genes of the H5N6 viruses isolated from the wild bird feces in Dhaka. The maximum likelihood phylogenetic tree was inferred, using IQ-TREE version 1.68, with 1000 bootstraps. The viruses identified in this study are shown in red. viruses from Bangladesh are marked in green; viruses from Komsomolsk-na-Amure are marked in blue; viruses from South Korea are marked in purple. Bootstrap values ≥70 are shown on branches.

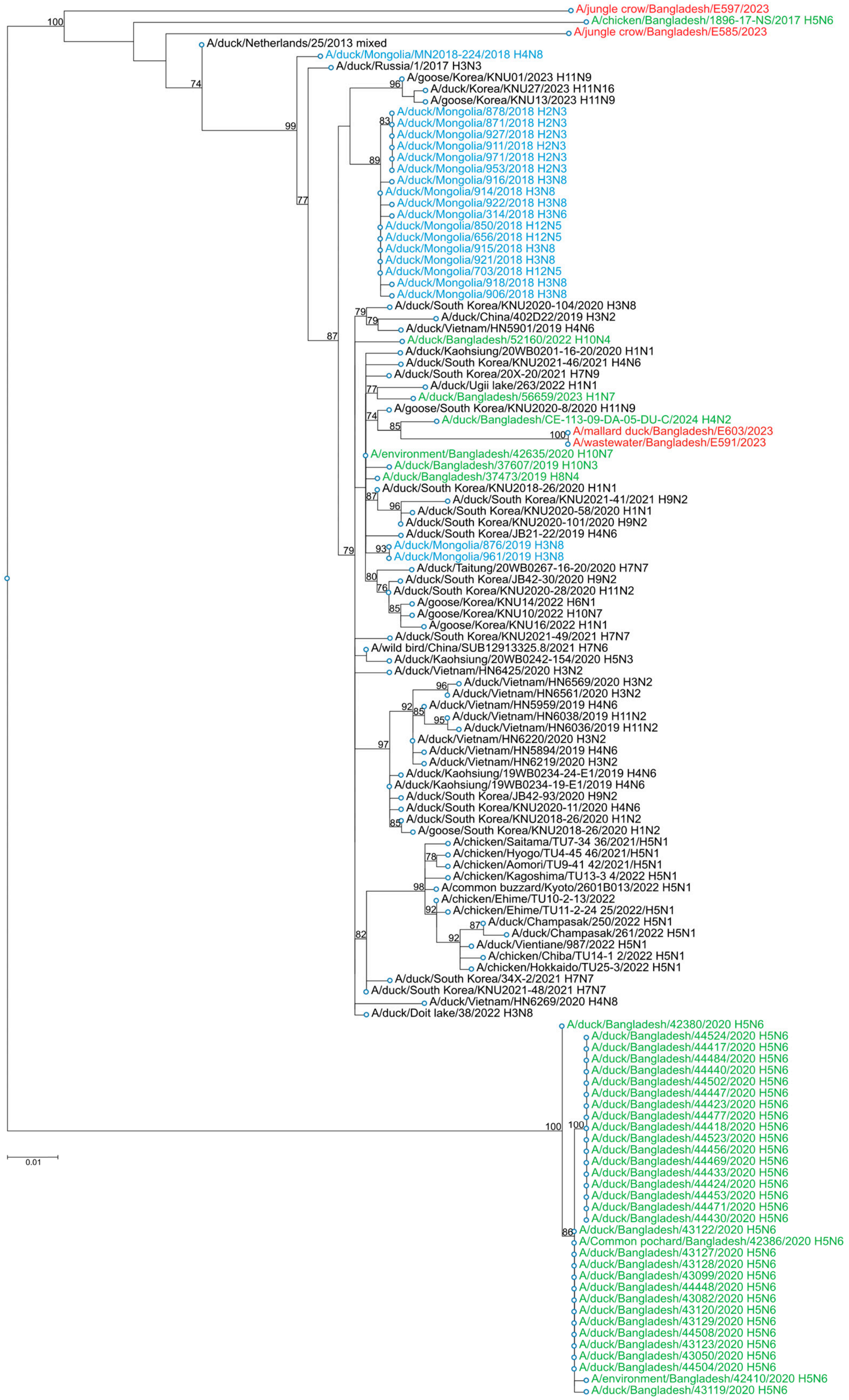

Figure S8 Phylogenetic relationship of the NS genes of the H5N6 viruses isolated from the wild bird feces in Dhaka. The maximum likelihood phylogenetic tree was inferred, using IQ-TREE version 1.68, with 1000 bootstraps. The viruses identified in this study are shown in red. Bootstrap values ≥70 are

shown on branches.

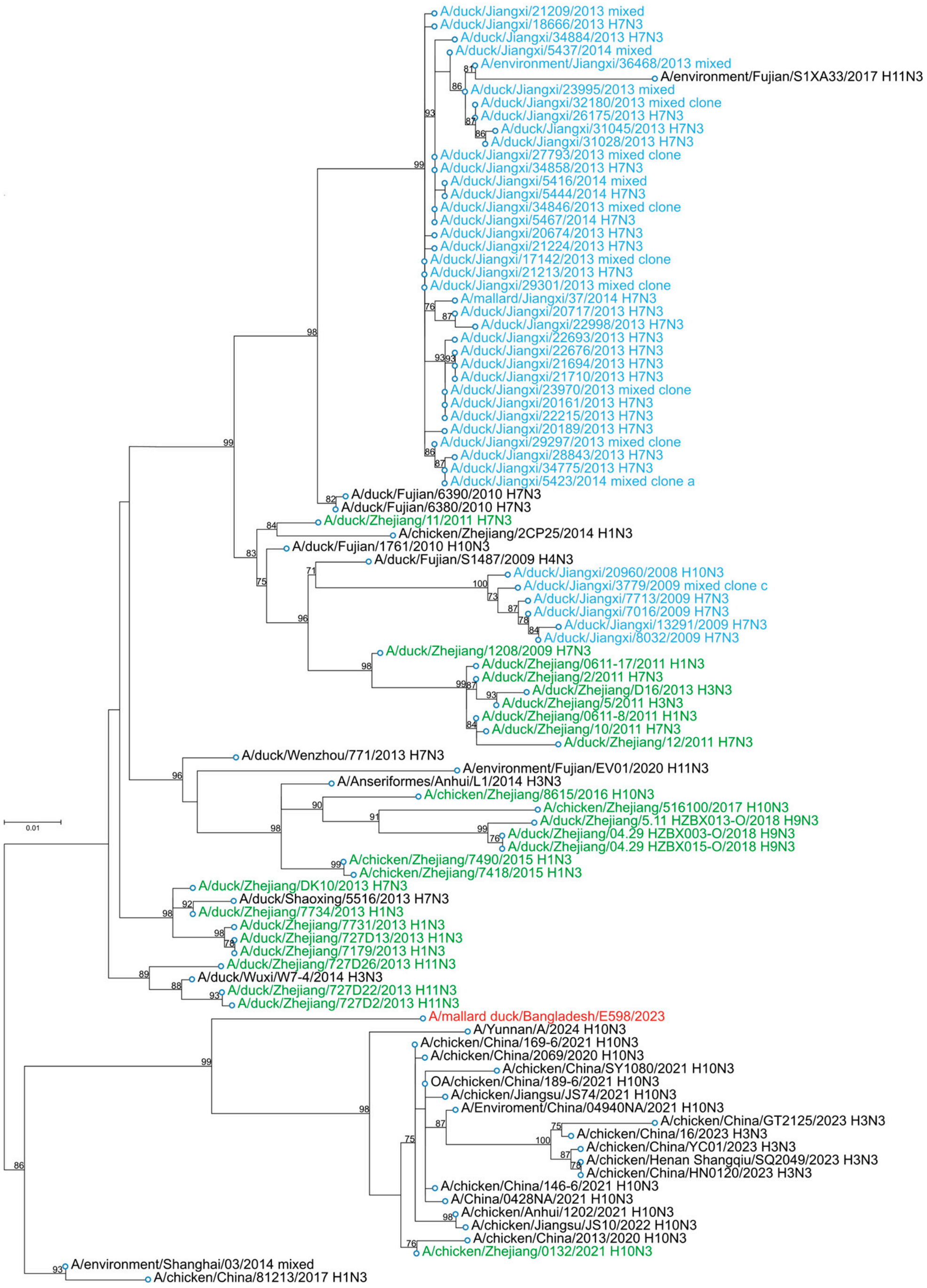

Figure S9 Phylogenetic relationship of the NA genes of the H1N3 viruses isolated from the wild bird feces in Dhaka. The maximum likelihood phylogenetic tree was inferred, using IQ-TREE version 1.68, with 1000 bootstraps. Influenza A viruses from this study are marked in red; viruses from Jiangxi, China are marked in blue; viruses from Zhejiang, China are marked in green. Bootstrap values ≥70 are shown on branches.

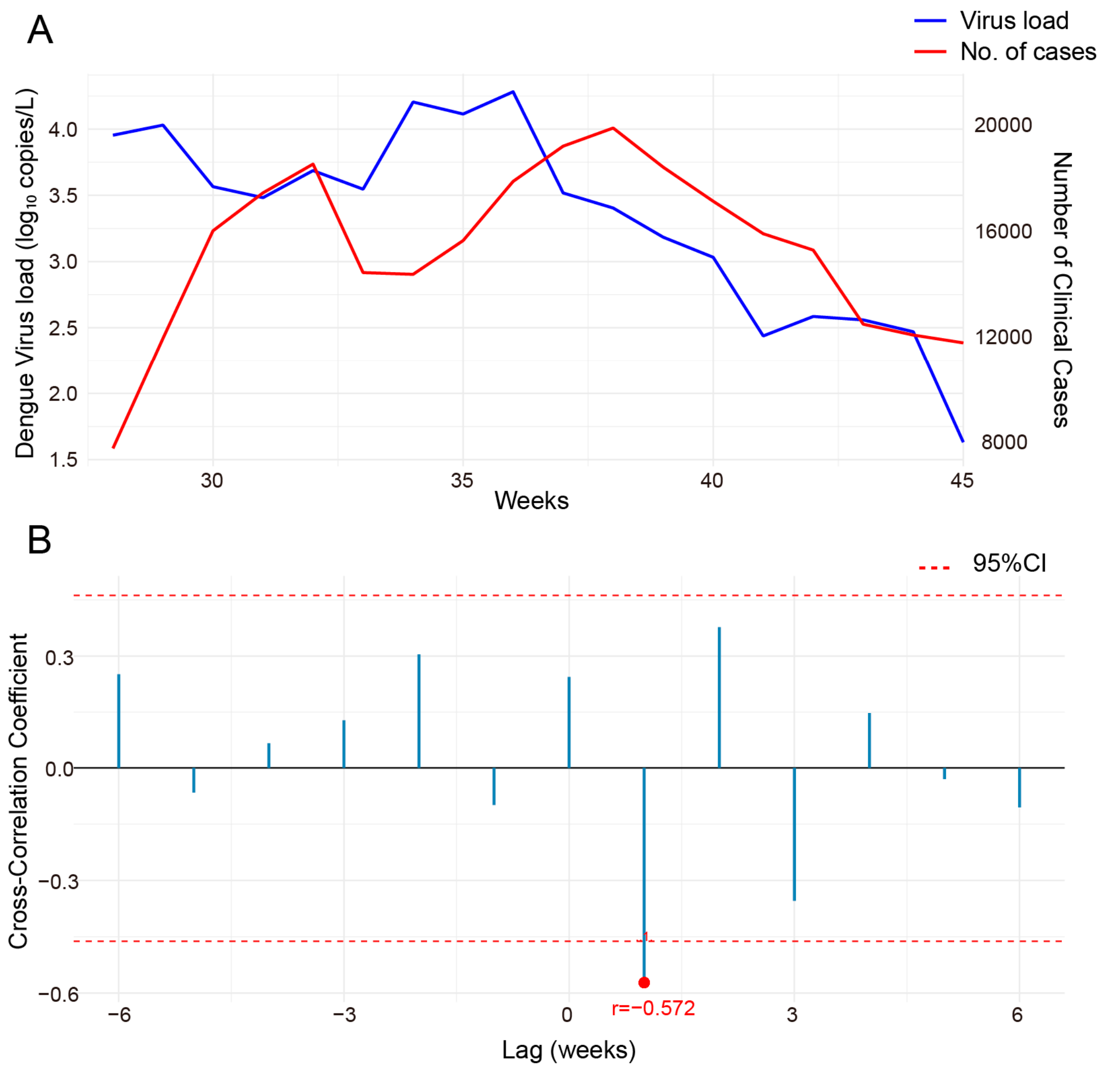

Figure S10. Temporal relationship between surface water dengue virus detection and clinical surveillance in Dhaka, Bangladesh. A: Time series of average dengue virus RNA concentration in surface water (blue line, left y-axis) and clinically reported dengue cases (red line, right y-axis) from epidemiological weeks 28-45, 2023; B: Cross-correlation function (CCF) analysis between surface water viral load and clinical cases. The dashed red lines indicate the 95% confidence interval (±0.462).

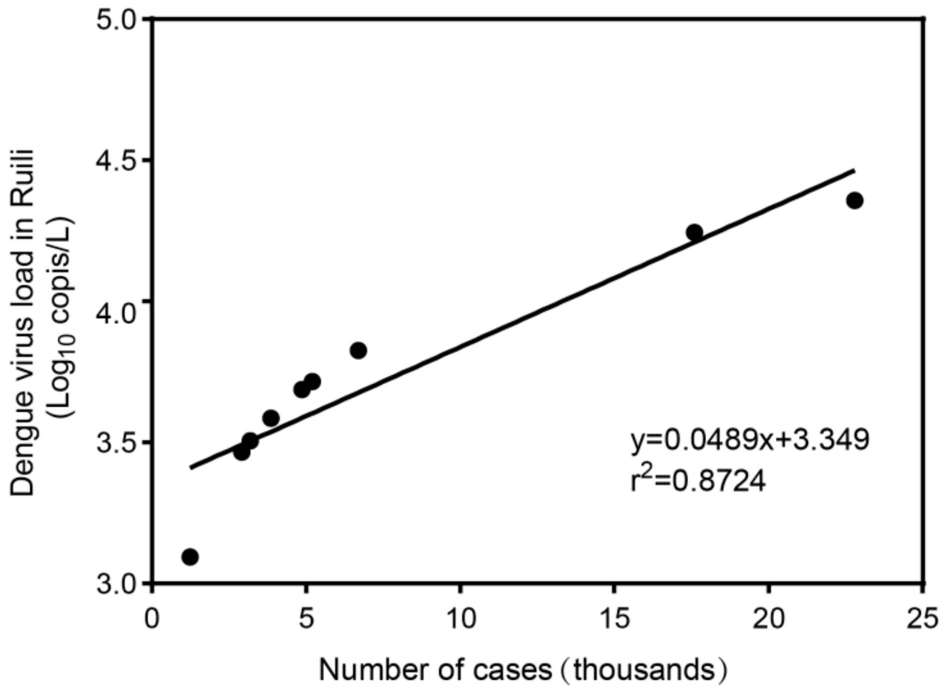

Figure S11 Regression analysis between DENV load in surface water and clinical cases in Ruili City. A significant linear relationship was observed between dengue virus load in the Ruili River and the number of weekly clinical cases. The linear regression equation is $y = 0.0489x + 3.3492$, with a coefficient of determination (r^2) of 0.8724.

Supplementary Table

Table S1. Real-time PCR primers and probes of two viruses.

Virus Type	Target gene	Oligo name	Sequence (5'-3')
AIV	M	AIV-M-F	GACCRATCCTGTCACCTCTGAC
		AIV-M-R	AGGGCATTYTGGACAAAKCGTCTA
		AIV-M-P	FAM-TGCAGTCCTCGCTCACTGGGCACG-BHQ1
AIV	H1	AIV-H1-F	CAGGTTATGGCACTGTCACGAT
		AIV-H1-R	CCATCTGCAGCAACACCATCTC
		AIV-H1-P	FAM-CTCTCCGAGAACAGGCCTCGACTTCAA–BHQ
AIV	H5	AIV-H5-F	AATGGGCACYTATCAGATACTATCA
		AIV-H5-R	GGARCACATCCADAAAGAYAGACCAG
		AIV-H5-P	FAM-TCAACAGTRGCRAGTTCCCTAGCAC-BHQ1
AIV	H7	AIV-H7-F	TTTGGTTTAGCTTCGGGGCATCA
		AIV-H7-R	AATAGTGCACCGCATGTTTCC
		AIV-H7-P	Cy5-ATGAARACAAGGCCCATTGCAATGGC-BHQ2
AIV	H9	AIV-H9-F	CTAGAAAGGCAGAAAATAGAAGG
		AIV-H9-R	ATATACAAATGTTGCATCTGCARG
		AIV-H9-P	Texas Red-CCATTTATTCGACTGTYGCCTCATC-BHQ1
AIV	H10	AIV-H10-F	CACAGTACAGAGAAGAAGC
		AIV-H10-R	ATATACAAATGTTGCATCTGCARG
		AIV-H10-P	FAM-TCAACAGTRGCRAGTTCCCTAGCAC-BHQ1
DENV	CprM	DENV-CprM-F	TTGTCCTAATGATGCTAGTCG
		DENV-CprM-R	AAACTGTCTCACTCGTAATCA
		DENV-CprM-P	ROX-ATGGTAAGTCAGTGACATTTACTGTGAC-BHQ2

Table S2. Standard curve of M, H1, H5, H7, H9, and H10 gene of avian influenza viruses (AIV) and CrpM gene of dengue virus (DENV).

Virus	Target gene	Standard curve	R2	Efficiency (%)	LOD Log10(copies/reaction)
AIV	M	y =-3.06x + 41.03	0.994	99.3	1.2
	H1	y=-3.34x + 43.26	0.996	99.7	1.1
	H5	y=-3.31x + 40.98	0.998	98.7	1.1
	H7	y=-3.39x + 39.33	0.991	99.8	1.1
	H9	y=-3.42x + 40.33	0.993	97.6	1.1
	H10	y=-3.32x + 39.93	0.999	99.5	1.1
DENV	CprM	y=-3.46x + 41.53	0.985	99.8	1.5

Table S3 Summary of sequencing data quality metrics.

Sample Name	Raw Data Q30	GC Conten t	Number of Raw Reads	Number of Clean Reads	Clean Reads Ratio	Mapping Coverage
Influenza_A_virus_A/wast ewater/Bangladesh/E591/2	93.55%	44.43%	5,435,798	5,342,704	98.29%	85.00%
Influenza_A_virus_A/mall ard	94.26%	45.24%	4,309,076	4,194,094	97.33%	84.55%
Influenza_A_virus_A/jung le crow/Bangladesh/E585/	94.22%	46.98%	5,508,594	5,428,212	98.54%	90.80%
Influenza_A_virus_A/jung le_crow/Bangladesh/E597/	92.73%	53.49%	9,287,124	9,154,752	98.57%	85.24%

Table S4 General information of sequence data of AIV.

Strain Name	Gene name	Type	Completeness	Accession
Influenza_A_virus_A/wastewater/Bangladesh/E591/2023	PA	/	53.52%	NA
Influenza_A_virus_A/wastewater/Bangladesh/E591/2023	HA	H5	95.39%	PV622343
Influenza_A_virus_A/wastewater/Bangladesh/E591/2023	NP	/	88.75%	PV627868
Influenza_A_virus_A/wastewater/Bangladesh/E591/2023	NA	N6	98.84%	PV627869
Influenza_A_virus_A/wastewater/Bangladesh/E591/2023	M	/	99.61%	PV627870
Influenza_A_virus_A/wastewater/Bangladesh/E591/2023	NS	/	99.89%	PV627871
Influenza_A_virus_A/mallard duck/Bangladesh/E603/2023	PB2	/	100.00%	PV635181
Influenza_A_virus_A/mallard duck/Bangladesh/E603/2023	PB1	/	68.43%	NA
Influenza_A_virus_A/mallard duck/Bangladesh/E603/2023	PA	/	99.82%	PV635182
Influenza_A_virus_A/mallard duck/Bangladesh/E603/2023	HA	H5	98.99%	PV635184
Influenza_A_virus_A/mallard duck/Bangladesh/E603/2023	NP	/	99.30%	PV635185
Influenza_A_virus_A/mallard duck/Bangladesh/E603/2023	NA	N6	97.61%	PV635186
Influenza_A_virus_A/mallard duck/Bangladesh/E603/2023	M	/	100.00%	PV635187
Influenza_A_virus_A/mallard duck/Bangladesh/E603/2023	NS	/	100.00%	PV636433
Influenza_A_virus_A/jungle_crow/Bangladesh/E585/2023	PB2	/	87.01%	PV636435
Influenza_A_virus_A/jungle_crow/Bangladesh/E585/2023	PB1	/	43.10%	NA
Influenza_A_virus_A/jungle_crow/Bangladesh/E585/2023	PA	/	29.87%	NA
Influenza_A_virus_A/jungle_crow/Bangladesh/E585/2023	HA	H5	96.40%	PV636436
Influenza_A_virus_A/jungle_crow/Bangladesh/E585/2023	NP	/	98.98%	PV636466
Influenza_A_virus_A/jungle_crow/Bangladesh/E585/2023	NA	N6	94.47%	PV636467
Influenza_A_virus_A/jungle_crow/Bangladesh/E585/2023	M	/	91.33%	PV636479

Influenza_A_virus_A/jungle_crow/Bangladesh/E585/2023	NS	/	93.71%	PV636480
Influenza_A_virus_A/jungle_crow/Bangladesh/E597/2023	M	/	98.25%	PV636490
Influenza_A_virus_A/jungle_crow/Bangladesh/E597/2023	NS	/	99.89%	PV636494
Influenza_A_virus_A/mallard duck/Bangladesh/E598/2023	NA	N3	68.07%	NA
Influenza_A_virus_A/mallard duck/Bangladesh/E598/2023	HA	H1	38.20%	NA

Table S5 Summary of Virus Subtypes Identified in Surface Water-Based Surveillance (SWBS) and Clinical Cases, and Their Phylogenetic Relatedness.

Location	Virus	Subtype in SWBS	Subtype in Clinical Cases	Phylogenetic Distance/Evidence for Phylogenetic Relatedness
Dhaka, Bangladesh	AIV	H5N6	/	SWBS sequence clustered with wild bird-derived H5N6 sequences (Fig. 1C).
Dhaka, Bangladesh	Dengue Virus	DENV-2	DENV-2 (68.1%)	SWBS sequences (e.g., DM33, DM35) formed a monophyletic clade with a clinical DENV-2 strain (MMC-RB-66) from Dhaka (GenBank: PP704405.1), indicating circulation of the same viral lineage (Fig. 2B).
Ruili, China	Dengue Virus	DENV-1	DENV-1	SWBS sequences clustered within a clade of DENV-1 clinical strains from Yunnan and Guangzhou, China, isolated in 2023, supporting local transmission of this lineage (Fig. 2D).

Table S6. Correlation with wastewater concentrations of dengue virus within 13 Baidu indexes.

X	Y	Intercept	Slope	r2	p
Dengue fever	sewage concentration	-166	1.7	0.694	<0.001
High fever	sewage concentration	-499	1.7	0.577	<0.001
Pain behind the eyes	sewage concentration	-47	2.6	0.478	<0.001
Rash	sewage concentration	44	4	0.476	<0.001
Severe headache	sewage concentration	-267	0.4	0.469	<0.001
Dengue hemorrhagic fever	sewage concentration	15	2.1	0.176	<0.001
Swollen glands	sewage concentration	26	2	0.111	0.001
Dengue virus	sewage concentration	74	5.1	0.106	0.001
Dengue shock syndrome	sewage concentration	55	1.6	0.064	0.017
mosquito	sewage concentration	94	0.1	0.07	0.726
Nausea/vomiting	sewage concentration	164	0	0.064	0.575
Joint pain	sewage concentration	149	-1.6	0.052	0.651
muscle pain	sewage concentration	126	0.1	0.031	0.657

Table S7 Using LM model to screen Baidu index related to wastewater concentration.

Index	Y	Intercept	Slope	r2	p
Dengue fever	sewage concentration	3120	3.8	0.691	1.61E-05
High fever	sewage concentration	3003	2.3	0.647	0.000256
Pain behind the eyes	sewage concentration	808	2.5	0.581	3.85E-02
Rash	sewage concentration	3003	1.8	0.51	0.0256
Severe headache	sewage concentration	2360	1.6	0.44	0.0821

Table S8 Using RF model to screen Baidu index related to wastewater concentration.

Index	%IncMSE	p value of %Inc MSE	IncNode-Purity	p value of IncNode-Purity	Percentage 1	Percentage 2
Dengue fever	12.136	0.01	1.21951E+12	0.01	0.081	0.136
High fever	11.182	0.01	1.13334E+12	0.01	0.064	0.026
Rash	12.023	0.01	8.35138E+11	0.01	0.048	0.04
Severe headache	6.398	0.01	3.61352E+11	0.04	0.039	0.043
Pain behind the eyes	4.599	0.03	3.48343E+11	0.02	0.03	0.045

Table S9 Using XGB model to screen Baidu index related to wastewater concentration.

Index	IMP_median	IMP_mean	IMP_num0	Color
Dengue fever	66613	86613	161	50000+
High fever	30366	42466	177	10001-50000
Rash	18771	28571	60	10001-50000
Severe headache	3121	16422	356	10001-50000
Pain behind the eyes	3036	5139	207	2501-5000
